# Nine‐year trend of oral anticoagulant use in patients with embolic stroke due to nonvalvular atrial fibrillation

**DOI:** 10.1002/joa3.12402

**Published:** 2020-07-20

**Authors:** Chisa Asahina, Ken Umetani, Keita Sano, Toshiaki Yano, Shin Nakano

**Affiliations:** ^1^ Department of Cardiology Yamanashi Prefectural Central Hospital Kofu Japan; ^2^ Department of Neurosurgery in Yamanashi Prefectural Central Hospital Kofu Japan

**Keywords:** anticoagulant, atrial fibrillation, DOAC, embolic stroke, nonvalvular

## Abstract

**Background:**

Direct oral anticoagulants (DOACs) are increasingly used as an alternative to warfarin in patients with nonvalvular atrial fibrillation (NVAF). However, whether there is sufficient prescription of oral anticoagulants (OACs) to decrease the incidence of embolic stroke remains unclear.

**Methods and Results:**

We conducted a retrospective observational study of patients hospitalized with ischemic stroke between January 1, 2010 and December 31, 2018. During the 8 years, the annual incidence ratio of embolic stroke to all ischemic strokes did not decrease over time (21‐33%) except for that in 2018. The proportion of OAC users did not also change over time (from 23% to 45% [overall 31%], *P* = .78). Among the OAC users, 19% patients were warfarin users, and 12% patients were DOAC users. In 73% of warfarin users, prothrombin time was subtherapeutic, whereas in 60% of DOAC users, the dose was adequately prescribed. OACs were prescribed more often in patients with high CHADS2 score than in those with low score (*P* = .01). The number of patients who had no medical history of a doctor visit before admission increased significantly in the recent period of 2015‐2018 (22% vs 8% in the previous period of 2010‐2014) (*P* = .01).

**Conclusions:**

The incidence of embolic stroke patients without OACs did not decrease over time, and OACs in patients with NVAF have not been sufficient, even in DOAC era. In recent years, the incidence of undiagnosed AF has increased. To prevent embolic stroke, a correct AF diagnosis beforehand is important.

## Graphical abstract

Embolic stroke owing to nonvalvular atrial fibrillation (NVAF) has not decrease over time, and oral anticoagulants have not been enough in NVAF patients, even in direct oral anticoagulant era. In recent years, the number of AF patients without a definitive AF diagnosis has increased. To prevent embo‐stroke, a correct AF diagnosis before embolic stroke is important. (Group1; had no history of medical institute. Group2; had no diagnosis of AF even with a periodic medical examination. Group3; had no OAC prescription for some reasons despite an AF diagnosis.)

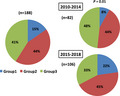

## 1 INTRODUCTION

Atrial fibrillation (AF) is a common cardiac arrhythmia that occurs most frequently in the elderly, and is associated with an increased risk of stroke and death.^1^, ^2^, ^3^ As average life expectancy continues to increase, the incidence of AF and AF‐associated embolic stroke is also expected to increase. Therefore, much attention has been focused on stroke prevention in patients with AF.

Warfarin has been used as the standard oral anticoagulant (OAC) to prevent embolic stroke and decrease mortality in patients with AF. In spite of these benefits, both general physicians and cardiologists have been hesitant to prescribe warfarin as primary prevention therapy^4^, ^5^, ^6^, ^7^ because of the increased risk of bleeding or intracranial hemorrhage. Even when warfarin is prescribed, it is often given at low dose, resulting in a subtherapeutic Prothrombin Time‐International Normalized Ratio (PT‐INR).^6^ This inappropriate use is one of the major issues with warfarin therapy in real‐world clinical practice. Direct oral anticoagulants (DOACs) have been introduced and are currently recommended by various guidelines as first‐line therapy for the prevention of embolic stroke in patients with nonvalvular AF (NVAF).^1^, ^2^, ^3^ DOACs are considered to have a better safety and efficacy profile, and are easier to use than warfarin. The overall proportion of OAC use has increased following the introduction of DOACs.^8^, ^9^, ^10^ However, whether embolic stroke has decreased since DOACs were launched remains unclear.

The aim of this study was to determine the annual use of OACs in patients with embolic stroke over 9 years, and to clarify the clinical problems with OAC use to prevent embolic stroke in these patients in the DOAC era.

## 2 METHODS

### 2.1 Patients

**Figure 1:**
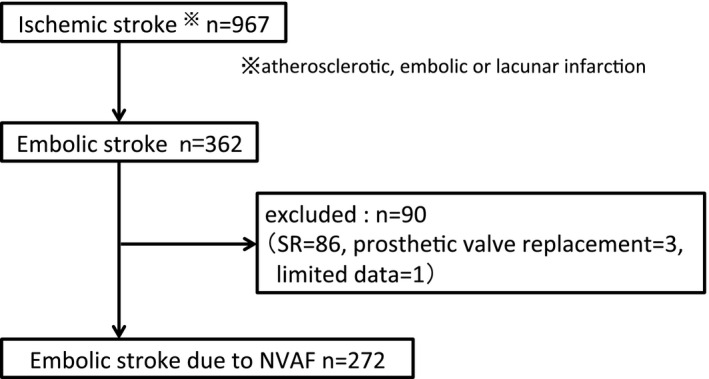
Flow diagram of data inclusion: 967 patients were hospitalized in the neurosurgical department because of ischemic stroke. Among these, 362 had been diagnosed with embolic stroke. Data from the remaining 272 patients with embolic stroke and NVAF were included. NVAF, nonvalvular atrial fibrillation; SR, sinus rhythm

In this retrospective observational study, we evaluated data from 967 patients with ischemic stroke hospitalized in the neurosurgical unit of Yamanashi Prefectural Central Hospital between January 1, 2010 and December 31, 2018. Most patients with stroke admitted to this neurosurgical unit because it is a regional core hospital. Ischemic stroke consisted of three types: atherosclerotic, embolic, or lacunar infarction. Each diagnosis was confirmed by our neurosurgical team based on clinical symptoms, neurological deficit, and computed tomography/magnetic resonance image findings. Among these patients, 362 (37%) presented with embolic stroke and their data were included in the study. The cardiac rhythm had been evaluated in all patients by a 12‐lead ECG and/or ECG monitoring, and was classified as either chronic atrial fibrillation (CAF), paroxysmal AF (PAF), or sinus rhythm (SR). CAF was defined as the presence of AF throughout the period of hospitalization. PAF was defined as a self‐terminating AF episode of >30 seconds. If both SR and AF were recorded during the hospitalization, the rhythm was classified as PAF. In the case of previously diagnosed AF, the rhythm was also classified as PAF, even if only SR was recorded during hospitalization. The rhythm was classified as SR if AF had not been previously recorded and was not observed during hospitalization. We excluded data from 90 patients (86 patients with SR, three patients with history of prosthetic valve replacement, and one patient because of limited data) (Figure 1).

We analyzed data from the remaining 272 patients (28%). The use of warfarin, DOACs or antiplatelet drugs (APDs) was confirmed based on medical record review. Activated partial thromboplastin time, PT‐INR and estimated glomerular filtration rate (eGFR) were obtained at the time of admission. The CHADS2 (Cardiac failure, Hypertension, Age, Diabetes, Stroke [Doubled]), CHA2DS2‐VASc (Congestive Heart failure, Hypertension, Age ≥75, Diabetes, Stroke [Doubled], Vascular disease, Age 65‐74, and Gender [female]), and HAS‐BLED (Hypertension, Abnormal renal/liver function [1 point each], Stroke, Bleeding history or predisposition, Labile INR, Elderly [>65 years], Drugs/alcohol concomitantly [1 point each]) scores were assessed. The Modified Ranking Scale (mRS) was used to classify the functional deficits determined by the neurosurgical team at hospital discharge. Echocardiography had been performed on 212 patients. In the other patients, echocardiography had not been performed or recorded because of their poor condition. Left ventricular (LV) end‐diastolic dimension, LV end‐systolic dimension, LV ejection fraction (LVEF), LV wall thickness, and left atrial dimension were obtained from two‐ dimensional or M mode echocardiography. An OAC was not prescribed for the following three reasons based on a review of medical records by two cardiologists, and these patients were divided into three groups: Group 1: no medical history of a doctor visit; Group 2: no prior diagnosis of AF, even when there was periodic medical follow‐up by a family doctor or hospital doctor; Group 3: other reasons to avoid OACs, even when there was a diagnosis of AF. To evaluate the use of OACs in patients with embolic stroke, and clarify the clinical problems of using OACs, several variables were compared annually, and between the previous (2010‐2014, 5 year) and recent (2015‐2018, 4 year) epochs.

### 2.2 Statistical analyses

Continuous variables are expressed as mean ± standard deviation. Categorical variables are expressed as number of patients and percentage. Differences between groups were tested for statistical significance using an unpaired Student's *t* test or Wilcoxon rank test based on distribution. We compared baseline categorical variables using a chi‐square test when appropriate, otherwise we used a Fisher exact test was used. *P* < .05 was defined as significant in tests of statistical inference. All statistical analyses were performed with IBM SPSS Statistics for Windows (version 21.0, IBM Corp.).

## 3 RESULTS

### 3.1 Clinical characteristics and the proportion of OAC users

**Figure 2:**
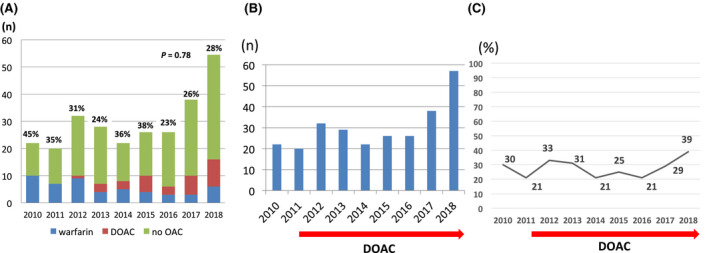
A, We have represented annual proportion of OAC users to all patients diagnosed with embolic stroke owing to NVAF on admission as a percentage at the top of each bar. Warfarin users decreased and DOAC users increased annually. However, there was no significant change in the proportion of OAC users. B, Trend of annual number of embolic stroke patients with NVAF. C, Trend of annual proportion of embolic stroke to all ischemic strokes (atherosclerotic, embolic, and lacunar). The proportion ranged between 21% and 33%. In 2018, the proportion of embolic stroke increased significantly to 39% (*P* = .02). DOAC, direct oral anticoagulant; NVAF, nonvalvular atrial fibrillation; OAC, oral anticoagulants

Of the 272 patients (146 male, 77 ± 10 years old) whose data we included (Figure 1). The patient characteristics are shown in Table 1. There were 207 (76%) patients with CAF and 65 (24%) with PAF. Any of OACs was prescribed in 31%; the annual proportion did not change significantly over time (*P* = .78) (Figure 2A). When the previous (2010‐2014, 5 years) and recent (2015‐2018, 4 years) periods were compared, the proportion of OAC use was not significantly different (34% vs 29%; *P* = .44). Warfarin was prescribed to 19% of patients, or a DOAC to 12%. Warfarin use decreased and DOAC use increased significantly (*P* < .01 for both, Figure 2A). Based on echocardiography, the left atrium was dilated ≥40 mm in 79% patients. A low LVEF (< 40%) was found in 6% of the patients. The mean CHADS2 score was 2.1 ± 1.3 and CHA2DS2‐VASc score was 3.5 ± 1.5. Embolic stroke was the cause of a poor prognosis as indicated by the mRS. During hospitalization, 30 patients (11%) died (mRS = 6). There were 155 (57%) patients (mRS ≥4), who were not able to return to usual daily life.

**Table 1:** Patient characteristics

Characteristic	Total (n = 272)
Age (y)	77 ± 10
<65	26 (10%)
65‐74	80 (29%)
≥75	166 (61%)
Male	146 (54%)
Rhythm	
CAF	207 (76%)
Oral anticoagulant	85 (31%)
Warfarin	51 (19%)
DOAC	34 (13%)
Antiplatelet drugs	56 (21%)
APDs without OAC	48 (18%)
Comorbidity	
Heart failure	70 (26%)
Hypertension	165 (61%)
Diabetes	46 (17%)
Stroke	58 (21%)
Vascular disease	13 (5%)
LAD (mm)	45 ± 8
<40 mm	45 (21%)
40‐50 mm	118 (56%)
>50 mm	49 (23%)
Ejection fraction <40%	13/212 (6%)
CHADS2 score	2.1 ± 1.3
0	29 (11%)
1	67 (24%)
2	81 (30%)
3	55 (20%)
≥4	40 (15%)
CHA2DS2‐VASc score	3.5 ± 1.5
0	8 (3%)
1	19 (7%)
2	47 (17%)
3	60 (22%)
4	64 (24%)
≥5	74 (27%)
HAS‐BLED score	1.5 ± 0.8
mRS score	
0‐1	40 (14.7%)
2‐3	77 (28.3%)
4‐5	125 (46%)
6	30 (11%)
eGFR (mL/min/min^2^)	
≥60	146 (5%)
30‐59	110 (40%)
≤29	16 (6%)

Abbreviations: APDs, antiplatelet drugs’ CAF, chronic atrial fibrillation; DOAC, direct oral anticoagulants; eGFR, estimated glomerular filtration rate; LAD, left atrial dimension; mRS, modified Ranking Scale.

### 3.2 Annual number of embolic strokes

The number of embolic stroke is shown in Figure 2B. The proportion of embolic stroke to all ischemic strokes in the patients did not alter significantly except in 2018; it has ranged from 21% to 33%, even after the clinical application of DOACs (Table 2, Figure 2C). In 2018, the proportion of embolic stroke increased significantly to 39% (vs that in 2017, *P* = .02). When the proportion of embolic stroke to all ischemic strokes between the previous and recent periods was compared, it was not significant different (27% vs 29%, *P* = .45).

**Table 2:** Annual number of embolic strokes

Year	Total (n)	Embolic stroke (NVAF) (n)	Others (n)^a^	Embolic stroke (NVAF)/total (%)
2010	73	22	51	30
2011	97	20	77	21
2012	96	32	64	33
2013	92	29	63	32
2014	105	22	83	21
2015	102	26	76	25
2016	121	26	95	21
2017	133	38	95	29
2018	148	57	91	39

Abbreviation: NVAF, nonvalvular atrial fibrillation.

^a^ Atherosclerotic, lacunar infarction, and non‐NVAF‐related embolic stroke.

### 3.3 OAC use in each CHADS2 score and age group

**Figure 3:**
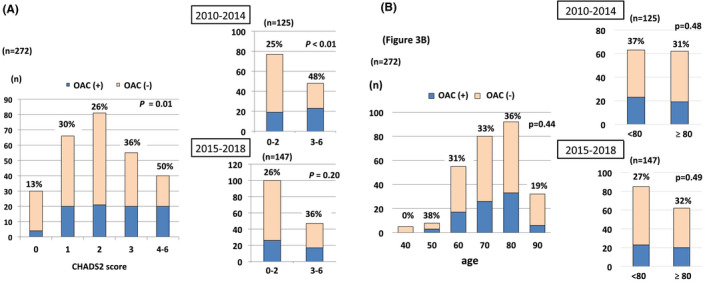
A, The proportion of OAC users to all patients diagnosed with embolic stroke owing to NVAF in each CHADS2 score category. This proportion was not significantly different in CHADS2 score range of 1‐3. When the low (0‐2) score and high (3‐6) CHADS2 score groups were compared, the proportion of OAC use was significantly higher in the high CHADS2 score group in overall (*P* = .01) and in the previous period (2010‐2014) (25% vs 48%, *P* < .01). This significant difference was not observed in recent period (2015‐2018) (*P* = .20). B, The proportion of OAC users to all patients diagnosed with embolic stroke owing to NVAF in each age group. Embolic stroke increased in the group ≥60 years old. The proportion of OAC use was not significantly different among each age group between 60 and 89 years old. In the recent period, this proportion decreased in the younger group and increased in the older group, but the difference was not significantly different. NVAF, nonvalvular atrial fibrillation; OAC, oral anticoagulants

The mean CHADS2 score was 2.1 ± 1.3 and CHA2DS2‐VASc score was 3.5 ± 1.5. The distribution of the CHADS2 score and the proportion of OAC use in each CHADS2 score group are shown in Figure 3A. The OAC use was at a similar proportion in the group with a CHADS2 score of 1‐3, but the use proportion was significantly greater in the group with a high (3‐6) score than in the group with a low (0‐2) score CHADS2 score (*P* = .01). The difference was significant in the previous period (48% vs 25%; *P* < .01), but not in the recent period (36% vs 26%; *P* = .20). Comparing the previous and recent periods, the proportion increased from 25% to 26% in the group with a low CHADS2 score and decreased from 48% to 36% in the group with a high CHADS2 score, but these differences were not significant. Embolic stroke occurred more frequently in elderly patients (61% of the patients were ≥75 years) (Figure 3B, Table 1). The proportion of OAC use was 31%‐36% in patients 60‐89 years old, and was not affected by age (*P* = .44). The proportion of OAC use was not different between patients aged <80 years and those aged ≥80 years (31% vs 31%, *P* = .95). When the previous and recent periods were compared, the proportion decreased in the younger group and increased in the elderly group, but the difference was not significant.

### 3.4 OAC use in patients with embolic stroke

**Figure 4:**
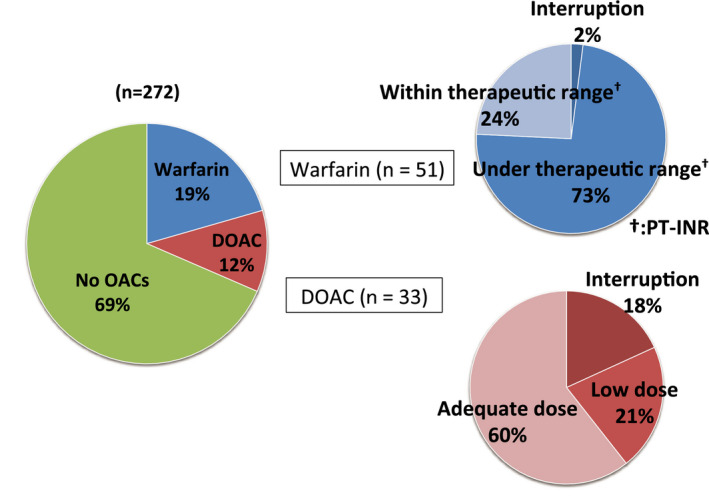
The proportion of OAC use in patients diagnosed with embolic stroke owing to NVAF. In 51 warfarin users, 73% of the patients PT‐INR were under therapeutic range. In 33 DOAC users, 60% of the patients were adequate dose users, and six patients (18%) had DOAC use interrupted for some reason. DOAC, direct oral anticoagulant; NVAF, nonvalvular atrial fibrillation; OAC, oral anticoagulants; PT‐INR, Prothrombin Time‐International Normalized Ratio

As shown in Figure 4, 51 (19%) patients used warfarin, and 33 (12%) patients used DOAC. The average PT‐INR values at the admission of the warfarin users were 1.47 ± 0.41. Of these, 37 (73%) patients had PT‐INR below the appropriate range according to the Japanese Circulation Society guidelines. DOAC use was interrupted 1 month before admission in six (18%) patients for the following reasons: two for gastrointestinal bleeding, two for prostate biopsy, one for low compliance, and one for unknown reason. In seven (21%) patients, the dose was low. Although an adequate DOAC dose was prescribed on admission in 20 (60%) patients, we could not confirm if the patients had adhered to their prescriptions based on their medical history.

**Figure 5:**
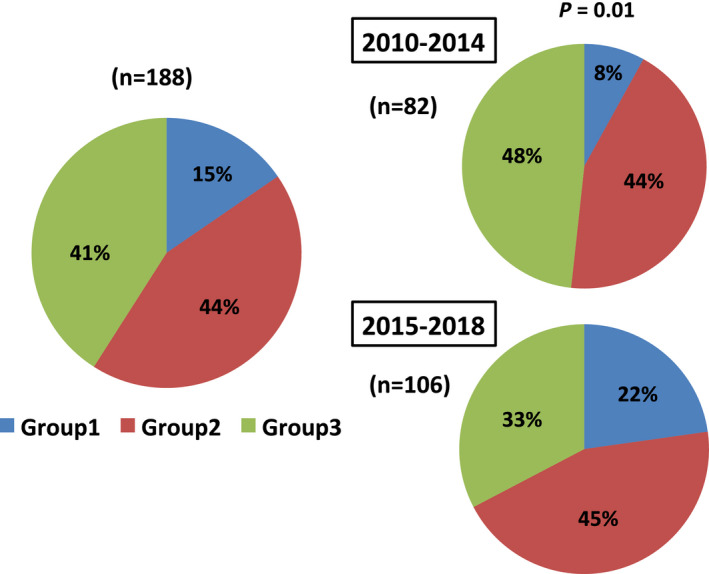
Embolic stroke patients without OAC on admission were categorized into three groups: Group 1; had no history of medical institute. Group2; had no diagnosis of AF even with a periodic medical examination, and Group 3; had no OAC prescription for some reason despite an AF diagnosis. In the recent period, the incidence of embolic stroke in patients who were not diagnosed with AF before admission (Group 1) increased significantly compared with the previous period (*P* = .01). AF, atrial fibrillation; OAC, oral anticoagulants

The remaining 188 patients (69% of the total patients), who were not prescribed an OAC, should have been prescribed an OAC, because 163 had a CHADS2 score ≥1 and 183 patients had a CHA2DS2‐VASc score ≥1. Overall, among 188 patients who had not given an OAC, Group 1 accounted for 15%, Group 2 for 44%, and Group 3 for 41%, respectively. When the previous and recent periods were compared, the proportion in Group 1 was significantly increased (*P* = .01) (Figure 5).

## 4 DISCUSSION

### 4.1 Major findings

This study demonstrates the clinical problems surrounding the prevention of embolic stroke in patients with NVAF. Over 9 years, even after the clinical introduction of DOACs, the ratio of embolic stroke to all ischemic strokes did not decrease. The incidence of embolic stroke in patients who were not treated with any type of OACs did not change. Around 70% of patients with embolic stroke were not prescribed OACs even in the DOAC era. AF was not diagnosed correctly in many patients with embolic stroke (59% of all embolic stroke patients without OAC) before admission, and this proportion increased in the recent period.

### 4.2 Annual number of embolic stroke patients

As average life expectancy is increasing, the incidence of AF and AF‐associated embolic stroke is also increasing. Figure 2B shows an annual increase in the number of embolic strokes in this study. In regional demographic statistics, the fraction of elderly (≥65 years old) in Yamanashi Prefecture increased from 23% to 29% in the recent decade (from 2008 to 2018), and this represents an increase of about 4000 people.^11^ The aging of the population in this area may account for the increased incidence of embolic events observed. This area has a basin topography and represents a localized area. Therefore, there was relatively limited population mobility during the study period. Our hypothesis is that the ratio of embolic stroke to all ischemic strokes could be reduced if OACs had been prescribed appropriately to all eligible patients with NVAF, because the most common cause of embolic stroke is NVAF.^1^ We also consider that embolic strokes should have been reduced in the DOAC era. However, our data suggest that the incidence and the proportion of embolic stroke did not decrease significantly, with the advent of the DOAC era.

The annual proportion of OAC users to all patients diagnosed with embolic stroke owing to NVAF on admission decreased from >30% to <30% (Figure 2A). If OACs had been prescribed adequately for eligible patients with AF, this proportion should have increased annually. Warfarin use decreased and DOAC use increased from 0% to 18%. This tendency reflects the widespread use of DOACs and is consistent with previous studies.^8^, ^9^, ^12^ However, the number of OAC users did not increase significantly. Therefore, the lack of a significant increase in the number of DOAC users over time may be one reason why the incidence of embolic stroke did not decrease. Many eligible patients with NVAF may have yet not to have OACs prescribed appropriately.

### 4.3 OAC use in each CHADS2 score and age group

The clinical usefulness of the CHADS2 score has been validated in previous reports and guidelines recommend its use in patients with NVAF.^1^, ^2^, ^3^, ^13^ This simple risk stratification score has been widely used in Japan. We observed that 11% of patients with embolic stroke were classified as having a CHADS2 score of 0, and only 13% of the patients in this group received an OAC (Figure 3A). The European Society of Cardiology guidelines proposed that the CHA2DS2‐VASc score is a better metric to identify low‐risk patients with NVAF. Patients with a CHA2DS2‐VASc score of 0 comprised only 3% of all patients with embolic stroke (Table 1). Patients with a CHADS2 score of 0 and CHA2DS2‐VASc score ≥1 should be prescribed OACs to prevent embolic stroke.^1^, ^3^ In the previous (2010‐2014) period, OACs were more often prescribed to those in the group with a high CHADS2 score (≥3) and were significantly underprescribed in low‐risk patients with a CHADS2 score 0‐2 (*P* < .01). This difference showed a relative decrease in the recent period. The recent widespread use of DOACs may be more evident in the group with a low CHADS2 score, as shown in previous reports.^8^, ^12^ However, DOACs should be used in all eligible patients with NVAF.

As reported previously, aging itself is a strong risk factor for ischemic stroke.^13^, ^14^ Embolic stroke events increased in patients ≥60 years old (Figure 3B). The average age of patients included in this study was 77 ± 10 years, which was the same as that in embolic stroke patients in a previous study,^12^ but was older than that (68‐73 years) in a previous large AF registry study.^6^, ^7^ To prevent and reduce strokes, the appropriate use of OACs in the elderly population should be considered. The widespread use of OACs in the elderly population (≥80 years old) is not obvious even in the DOAC era (Figure 3B). This finding indicates that a substantial number of eligible patients with high‐risk AF remain without OAC prescriptions.

### 4.4 OACs use in patients with embolic stroke

Our present data showed that only 31% of patients with embolic stroke had received OACs (Figure 4). Warfarin was used in 19% of patients and DOAC was used in 12% of patients. As in previous reports, warfarin control of most of the patients with embolic stroke was at a low dose in this study.^6^, ^7^ However, in the patients taking DOACs, 20 (60%) patients took an adequate dose. We could not confirm whether the patients ever forget to take their daily DOAC dose, and we could not confirm the presence of the drug from the blood coagulation data on admission.

Despite the widespread use of DOACs, it is not clear why the embolic stroke incidence has not decreased. This study revealed that AF was not diagnosed correctly on admission in 59% of the patients with embolic stroke (Group 1 and 2 in Figure 5). Furthermore, in the recent period, the ratio of embolic stroke in Group 1 increased significantly. In other words, correct use of OACs by patients with a diagnosis of AF may prevent embolic stroke and embolic stroke events have relatively increased in the patients without AF diagnosis. The lack of periodic ECG recording or asymptomatic AF events may be the reason. To reduce embolic strokes, it is important to prescribe OACs to eligible patients, and to diagnose AF correctly based on the ECG. To improve the recognition of AF and prevent embolic stroke it is useful to perform periodic pulse check or ECG recording, or use other modalities, such as a modern home sphygmomanometer or a wearable device to identify pulse irregularity.^15^

## 5 STUDY LIMITATIONS

There are several clinical limitations in this study. The present data came from a localized single center database and the population sample size is small. Because the study population was small, it was difficult to determine the actual annual change in the incidence of embolic stroke. To analyze the incidence rate of embolic stroke in patients with NVAF before occurrence of stroke, whether embolic stroke events were reduced, and whether OACs have been sufficiently prescribed to eligible patients with NVAF, we need to create and track a large‐scale registry of patients with NVAF in this area. In this study, we would like to clarify the issues of current anticoagulant therapy from patients with embolic stroke. The reasons for nonprescription of OACs before embolic events was underrecognition of AF, and in turn, reasons for underdiagnosis of AF were medical history, previous ECG or other information only based on medical records. There were not confirmed strictly the reasons for underdiagnosis of AF. Warfarin users should be evaluated by time in therapeutic range. However, because of the limited clinical information before the onset of embolic stroke, only PT‐INR value at the admission was used.

## 6 CONCLUSIONS

This study revealed the clinical problems surrounding the prevention of embolic stroke in patients with NVAF. The number of embolic strokes caused without OACs prescription has not been decreasing, and OACs have not been used sufficiently in eligible patients with NVAF, even in the DOAC era. In recent years, the number of AF patients without a definitive AF diagnosis has increased. This may be one of the reasons why OACs are not being prescribed to eligible patients with NVAF. To prevent embolic stroke, a correct AF diagnosis before stroke events and appropriate OAC use are important.

## DISCLOSURES

No authors have any potential conflict of interest to declare in relation to the present work.
